# IFITM proteins: Understanding their diverse roles in viral infection, cancer, and immunity

**DOI:** 10.1016/j.jbc.2022.102741

**Published:** 2022-11-23

**Authors:** Maria Gómez-Herranz, Jordan Taylor, Richard D. Sloan

**Affiliations:** 1 International Centre for Cancer Vaccine Science, University of Gdańsk, Poland; 2 Institute of Genetics and Cancer (IGC), University of Edinburgh, Edinburgh, United Kingdom; 3 Infection Medicine, School of Biomedical Sciences, University of Edinburgh, Edinburgh, United Kingdom; 4 ZJU-UoE Institute, Zhejiang University, Haining, Zhejiang, China

**Keywords:** interferon, innate immunity, cancer, translation, antiviral factors, restriction factors, IFITMs, IFITM, IFITM1, IFITM2, IFITM3, HA, hemagglutinin, IAV, influenza A virus, IFITMs, interferon-induced transmembrane proteins, IFN, interferon, ISG, IFN-stimulated genes, MCMV, murine cytomegalovirus, TCR, T-cell receptor

## Abstract

Interferon-induced transmembrane proteins (IFITMs) are broad spectrum antiviral factors that inhibit the entry of a wide range of clinically important pathogens including influenza A virus, HIV-1, and Dengue virus. IFITMs are thought to act primarily by antagonizing virus–cell membrane fusion in this regard. However, recent work on these proteins has uncovered novel post-entry viral restriction mechanisms. IFITMs are also increasingly thought to have a role regulating immune responses, including innate antiviral and inflammatory responses as well as adaptive T-cell and B-cell responses. Further, IFITMs may have pathological activities in cancer, wherein IFITM expression can be a marker of therapeutically resistant and aggressive disease courses. In this review, we summarize the respective literatures concerning these apparently diverse functions with a view to identifying common themes and potentially yielding a more unified understanding of IFITM biology.

Cells are subjected to numerous events that affect their survival. The innate immune system is the first line of host cell defense, acting to recognize and eliminate threats. Interferons (IFNs) are pleiotropic cytokines of the innate immune system that confer antipathogen and antitumor properties to the host (1, 2). Their activities include shutting down host translation machinery, induction of an antiviral state, driving apoptosis (or immunogenic necroptosis), and activation of peripheral immune cells. IFN signaling is thus at the core of immune regulation, and to achieve its effects, it induces the expression of hundreds of IFN-stimulated genes (ISGs), which include IFN-induced transmembrane proteins (IFITMs) (3).

The human IFITM family consists of five proteins. Of these, IFITM1, IFITM2, and IFITM3 are expressed ubiquitously and are inducible by type I, type II, and type III IFNs due to the presence of IFN response elements (ISREs) and gamma-activated sequences (GASs) in their promoters (4, 5, 6, 7). Phylogenetic studies also associate IFITM5 and IFITM10 with the IFITM family in humans (8). IFITM5 and IFITM10, however, are not IFN inducible and have not been implicated in host defense and so will not be examined in this review. The human IFITM gene cluster is located on chromosome 11 (9). Orthologous genes are found in numerous vertebrates such as mice and nonhuman primates but also in marsupial, avian, and amphibian species (8, 10). In the main, all three antiviral IFITM proteins share a high sequence identity, however, the termini are more distinct: IFITM1 has an extended C terminus with additional 13 amino acids and IFITM2 and IFITM3 have extended N termini with an extra 20 and 21 amino acids, respectively (10).

The IFITM genes were discovered and characterized in the 1980s by the Stark and Kerr laboratories (5, 11, 12). Since then, substantial research has focused on exploring their role in blocking viral infection (13, 14). Viruses are highly diverse, and their rapid evolutionary rates compromise the efficacy of cellular antiviral mechanisms. Suppression of a broad spectrum of viruses by innate immunity thus presents an evolutionary challenge. Nonetheless, IFITMs are able to restrict viruses from a variety of taxa. For example, human IFITMs repress replication of influenza A virus (IAV) and flaviviruses such as dengue virus and tick borne encephalitis virus, as well as HIV-1 and SARS coronavirus (SARS-CoV) (15, 16, 17, 18, 19). Functional studies of IFITM orthologs in mice and macaques show that IFITMs protect against virus infection in nonhuman species and *in vivo* (20, 21, 22). To date, most studies implicate IFITMs in the blockade of viral entry; however, emerging work suggests that they also act at later stages of viral replication cycles (23, 24).

Recent work has also identified activities of IFITMs beyond viral restriction, notably in the context of innate immunity and cancer. Numerous studies point to anti-inflammatory activities of IFITMs, while in cancers, elevated expression of IFITMs has been associated with poor therapeutic outcomes (25, 26). The latter adds IFITMs to a growing list of antiviral and immunoregulatory ISGs with dual roles as players in cancer biology (27). It is useful to consider the origins of this duality, as it offers a paradigm that may help clarify disparate strands in the extant IFITM literature.

Viral infection and cancers exhibit important similarities. Both malignant and infected cells are fundamentally hostile to and molecularly distinct from the host. Both typically exhibit altered antigen repertoires, rendering them subject to killing by cytotoxic T cells (28, 29), or downregulate MHC class I expression, rendering them subject to killing by natural killer cells (30). Both prime innate immunity through overlapping nucleic acid detection pathways (31, 32) and both trigger overlapping pathways of programmed cell death (33, 34). More pointedly, viruses themselves account for an estimated 15% of all cancers worldwide (35) and such “oncoviruses have critically informed modern understanding in the molecular biology of cancer (36, 37). Additionally, even in cancers of noninfectious origin, reactivation of endogenous retroviruses is a common feature (38, 39) that promotes tumor genome instability (40) and facilitates epithelial to mesenchymal transition (a hallmark of cancer) (41, 42).

Thus, cellular and etiological commonalities between viral infection and tumorigenesis have given rise to overlapping host response mechanisms, frequently driven by ISGs, which can promote both viral and tumor clearance (43, 44, 45). Concomitantly, in excess, those ISGs which act as negative feedback regulators can also promote adverse outcomes (46, 47, 48). It is our contention that viewing IFITMs in light of this unified host-response paradigm may prove fruitful. To that end, in this review, we seek to bring together hitherto separate literatures on the IFITMs with a view to facilitating such a holistic perspective.

## The role of IFITMs in viral infection

Considerable *in vivo* evidence supports the importance of IFITMs in viral infection control, particularly in the case of IFITM3. Mice lacking *Ifitm3* are more susceptible to infection by IAV, West Nile Virus (WNV), and a range of alphaviruses (20, 49). In humans, the IFITM3 allele rs12252-C correlates with a higher risk of severe IAV infection and hospitalization (21, 50). The same polymorphism has been linked to a more severe course of acute HIV-1 infection (51) and worse COVID-19 outcomes due to SARS-CoV-2 infection (52). However, whether this variant is truly causal or by what mechanisms it may act, remains controversial (53). Another variant in the IFITM3 promoter, rs34481144, reduces binding of the transcription factor IRF3 and is associated with increased susceptibility to IAV infection in patients (54) (for a complete review on IFITM polymorphisms in humans see Zhao *et al.* (55)).

**Figure 1:**
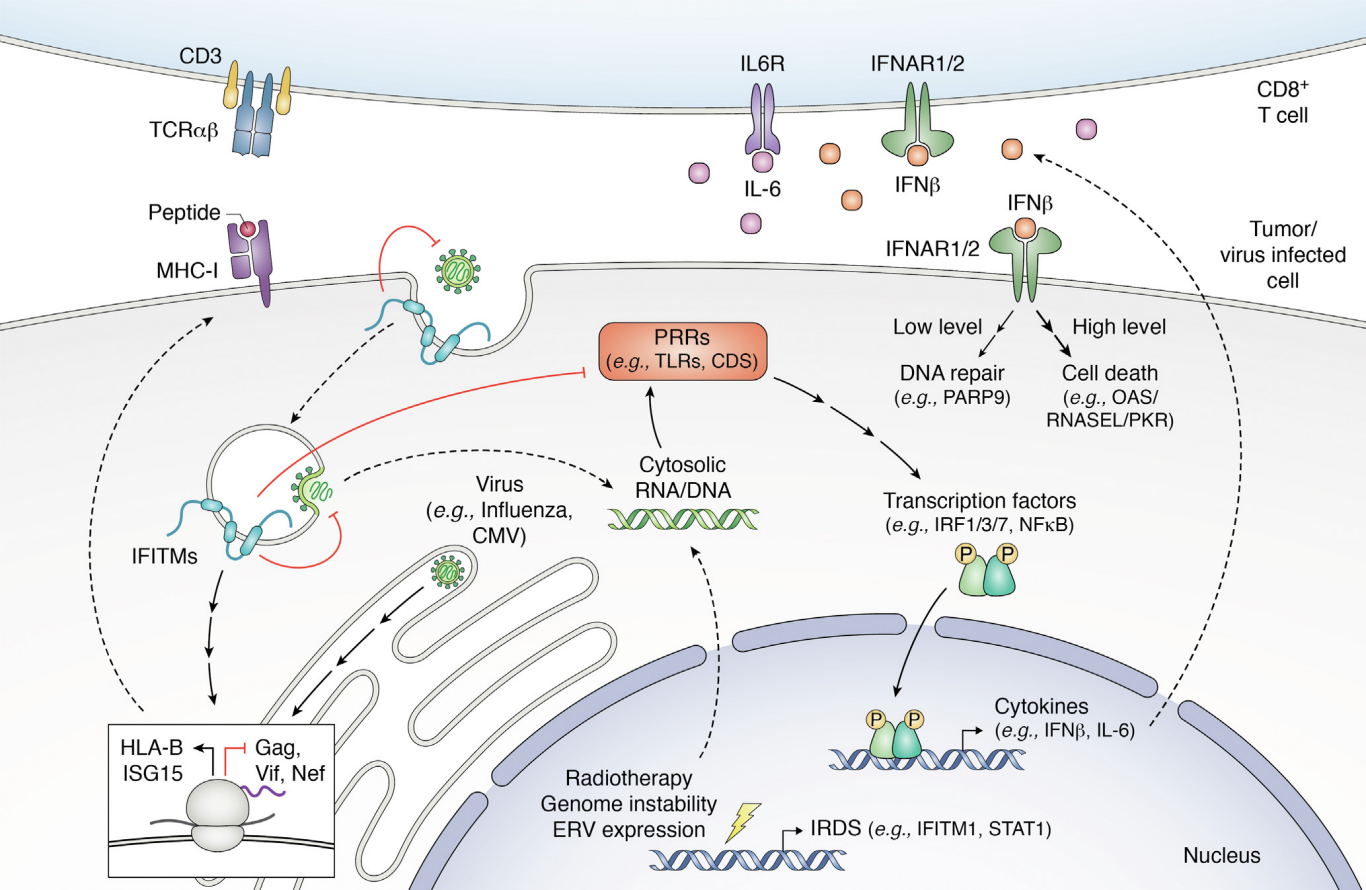
**The diverse roles of IFITM proteins in infected cells and tumor cells.** IFITM proteins (depicted in *blue*) are predominantly anchored to the endolysosomal and plasma membrane compartments where they are involved in multiple cellular processes including the canonical inhibition of virus entry. IFITMs also regulate the translation of targeted proteins such as HIV-1 Gag but also host cell proteins such as HLA-B and ISG-15 cell proteins (depicted in the ribosome box) through as yet undefined mechanisms. In addition, IFITMs are increasingly thought to regulate innate immune activation as they have been shown to reduce type I IFN and IL-6 secretion. These proinflammatory immune responses may originate from viral pathogen-associated molecular patterns or be self-derived as a consequence of cancer-induced genome instability, endogenous retrovirus expression, or radiotherapy. A range of signaling processes have been suggested for this anti-inflammatory role, all of which ultimately reduce inflammatory gene expression. Altered levels of MHC class I in the target cell may influence CD8^+^ T-cell–mediated killing in response to oncopeptides, while the relative abundance of type I IFN signaling may determine if DNA repair is activated or cell death responses occur.

Mechanistically, IFITMs are thought to inhibit the cytosolic entry of viruses by altering the physical characteristics of membranes (Fig. 1) (50, 56, 57). IFITM3 is reported to interact with vesicle membrane protein-associated protein A (VAPA) and impede its association with oxysterol-binding protein (OSBP) (56). This may increase endosomal cholesterol content and thereby impede viral entry. These findings are consistent with the observation that the lipophilic antifungal drug amphotericin B could rescue IAV infection in IFITM3 overexpressing cells (58). Additionally, a novel N-terminal amphipathic helix motif was shown to be an important determinant of IFITM3-mediated restriction of a number of viruses, including IAV, Zika virus, and Ebola virus (59). The IFITM3 amphipathic helix was shown to induce negative membrane curvature (57), which is unfavorable for virus–cell membrane fusion, in a cholesterol-dependent manner. More recent work suggests this activity requires direct binding of cholesterol by the IFITM3 amphipathic helix (60, 61).

In addition to their canonical influence on viral entry, IFITMs may have distinct means to suppress infection, and these have been particularly studied in the case of HIV-1. In the first characterization of their anti-HIV-1 activity, IFITM1, IFITM2, and IFITM3 each independently restricted multicycle HIV-1 infection (18). However, only IFITM2 and IFITM3 were shown to restrict entry of the CXCR4-tropic virus used, whereas IFITM1 did not, despite otherwise restricting virus replication. This was the first suggestion that IFITM proteins might restrict viruses at replication steps distinct from virus entry. Other studies have suggested that IFITMs restrict viruses by distinct mechanisms, though these comprise a minority of the literature to date. For instance, IFITMs were shown to reduce the replication of HIV-1 by significantly downregulating the expression of viral proteins from transfected proviral complementary DNA, which in turn reduced levels of HIV-1 production (23, 24) (Fig. 1). IFITMs have also been found to interact with HIV-1 envelope glycoproteins (Env) and to disrupt their proteolytic processing (62). Similarly IFITM3 has been found to interact directly with IAV hemagglutinin (HA) (63), while interactions between IFITM proteins and SARS-CoV-2 spike have also been described and may influence infection outcome (64).

IFITMs can also be found in viral particles themselves, and their presence reduces infectivity by suppressing the ability to fuse to cellular membranes and consequently restricting entry into target cells (65, 66). This antiviral property, termed ‘negative imprinting’, is observed in a broad range of viruses including HIV-1, Ebola virus, and WNV (67). Negative imprinting might reasonably occur through a similar mechanism to how IFITMs disrupt virus–cell membrane fusion in target cells, that is, *via* altering virus membrane properties. One study noted that IFITM3 expression displaced IAV HA from viral particles, which in turn sensitized the virus to neutralizing antibodies (68). At present, it is unclear whether this mechanism involves direct binding between IFITMs and HA (63) or if it will be applicable to other viruses.

Overall, IFITMs are versatile restriction factors that are able to suppress viral infection. Principally, our understanding of them is through their influence on membranes to inhibit virus entry, yet many questions remain as to their underlying cell biology given the range of alternative antiviral mechanisms that have also been described.

## The role of IFITMs in immune regulation

The protective antiviral roles played by IFITM proteins have been demonstrated in mouse infection models and further supported by human molecular genetics. However, not all of their effects *in vivo* can be explained through direct antiviral restriction. Several studies observed elevated inflammatory signaling in IFITM-deficient models (21, 49, 69), but they were unable to disentangle these effects from differences due to increased viral entry. An elegant study from the Humphreys lab used a murine cytomegalovirus (MCMV) model to circumvent this issue (70). This study showed that MCMV is not restricted by IFITM3 but nevertheless noted inflammation-driven pathology and concomitant loss of viral control in *Ifitm*3-deficient mice. The mice had markedly increased IL-6, TNFα, and IFNα production in the course of infection. Notably, *ex vivo* cultured dendritic cells from IFITM3-deficient mice also exhibited exaggerated IL-6 production in response to endosomal Toll-like Receptor (TLR) agonists (CpG DNA and poly(I:C)), demonstrating convincingly that IFITM3 regulates cell-intrinsic innate immune signaling.

These data may contrast somewhat with more recent work providing evidence for a proinflammatory role of IFITMs in an allergic airway inflammation model (71). Yánez *et al.* observed reduced T helper (Th) 2-driven and TNFα/IL-6–mediated pathology, together with enhanced Th1/IFNγ responses in papain-sensitized mice lacking the entire IFITM locus (IFITMdel mice). This is notable in light of reports that *Ifitm* genes are transcriptionally repressed by *Bcl6* (72), which itself is required to control spontaneous Th2 inflammation in mice (73). *Bcl6* and *Ifitm3* are also both required for B-cell affinity maturation (74, 75), consistent with the general importance of a “BCL6-IFITM” pathway in immune function. Murine *Ifitm* genes are also transcriptional targets of the Hedgehog and Wnt signaling pathways (76, 77), which both appear to promote Th2 skewing (78, 79). Thus, whether IFITMs are proinflammatory or anti-inflammatory should depend substantially on which cell types drive inflammation in a given disease context.

In line with this idea, in a murine colitis model, *Ifitm3*-deficient mice exhibited enhanced Th17 driven pathology accompanied by reduced IFNγ/Th1 responses compared to WT mice (80). In the same study, IFITMdel mice developed spontaneous chronic colitis from an early age. Interestingly, a more recent study has also found that *Ifitm3*-deficient mice infected with *Listeria monocytogenes* exhibit enhanced production of both IL-12, a Th1-promoting cytokine, and IL-4, a Th2-promoting cytokine (81); however, Th cell subsets were not analyzed in detail. The enhanced immune priming in *Ifitm3*-deficient mice explained improved pathogen clearance in this model, thus providing the first *in vivo* evidence for the importance of immune regulation by IFITMs in a nonviral infection modality.

The balance between Th2-Th1 and Th1-Th17 T-cell phenotypes has emerged as a critical paradigm in the regulation of adaptive immunity and immune pathology in the last several decades (82). It is tightly regulated by a complex network of cytokines and also depends crucially on the mode of activation of antigen-presenting cells by ligation of innate receptors such as TLRs (83). MyD88-deficient mice with attenuated TLR signaling capacity exhibit extreme Th2 skewing behavior, for example (84). Thus, the negative regulation of innate TLR signaling in dendritic cells documented before could potentially account for some of the observed T-cell phenotypes. At the same time, T-cell phenotypes are also influenced by intrinsic factors such as T-cell receptor (TCR) signaling strength. It is well documented that stronger TCR signals bias differentiation toward a Th1-type pathway while weaker TCR signals promote Th2-skewed differentiation. In this regard, the finding by Lee (75) that IFITM3-deficient Jurkat cells exhibit attenuated TCR signaling may be significant; however, it predicts effects in T-cell skewing behavior opposite to those predicted by the findings in TLR signaling. Regardless, adoptive cell transfer and conditional gene targeting strategies will be required to clarify the cell type contributions to these phenotypes in a variety of disease models.

It seems likely that IFITM proteins harbor context-specific immunoregulatory activities. Detailed study of cell intrinsic signaling effects of IFITMs in distinct cell populations may be required to provide a clearer picture to reconcile the available data. On this matter, a recent preprint from the Humphreys group has provided new evidence in both murine and human dendritic cell models that IFITM3 regulates TLR internalization dynamics (85). Mechanistically, this is proposed to proceed by IFITM3-dependent turnover of a poorly studied innate immune signaling protein, Nogo-B.

In addition, other recent molecular studies have found indicative evidence that IFITM3 may regulate the cGAS-STING-IRF3 signaling pathway, which is not known to be regulated by Nogo-B. A protein–protein interaction screen for signal-dependent STING interactors discovered IFITM3 binds to STING during late phase signaling (86). An additional proteomic study uncovered p62/SQSTM1 as a strong candidate IFITM3 interactor (87). Work from the Paludan lab has shown that p62/SQSTM1 is phosphorylated by TBK1 and participates in a negative feedback loop on the cytosolic DNA sensing pathway by facilitating autophagic degradation of STING (88). Tantalizingly, another study has provided evidence that IFITM3 may promote the autophagic degradation of IRF3, leading to reduced type I IFN production upon Sendai virus infection or poly(I:C) treatment (89). These data may also be consistent with the observation that swine IFITM3 overexpression appears to regulate IFNα/IFNβ mRNA levels upon lipopolysaccharide treatment in a swine kidney cell line (90). Taken together, these findings imply a potentially general anti-inflammatory role for intracellular IFITM3 (other IFITM proteins were not investigated in these studies). It is significant that all four of the MCMV, experimental colitis, allergic airway inflammation, and *Listeria* models discussed previously have been shown to be critically impacted by cGAS-STING-IRF3 signaling (91, 92, 93, 94, 95, 96, 97, 98).

The STING pathway is also an important contributor to the development of an efficacious antitumor immune response (Fig. 1) (99, 100) and therefore to the efficacy of a variety of cancer therapies, including radiotherapy (48, 99, 101). Thus, the roles of IFITMs in cancer (discussed in detail in the following section) may also be fruitfully viewed in the light of the immunoregulatory actions of these proteins, thus far only investigated closely for IFITM3. The early study by Kita *et al.* (102) showing the functional involvement of IFITM1 in a pathway of IFNα-induced DNA damage resistance adds a further nuance to this interconnection. For these authors, the coincidence of immunomodulatory roles of the IFITMs and tumor-promoting roles is hard to ignore in the search for unifying hypotheses. It is tempting to speculate with respect to cancers that the IFITMs could potentially function in a broadly analogous manner to negative regulatory ISGs such as TREX1 and ADAR1, which negatively regulate antitumor immunity and thereby promote therapy resistance (Fig. 1) (46, 103).

## The role of IFITMs in cancer

Accumulating evidence is in support of IFITMs playing a role in tumor progression. The protein expression of IFITM1, IFITM2, or IFITM3 are predictors for poor prognosis in numerous cancers such as colorectal (104, 105, 106), prostate (107), ovarian (108), lung (109), liver (26), breast (7, 110), and astrocytoma (111). As such, IFITMs have been proposed as a prognostic biomarker for distinct types of cancer. For example, overexpression of IFITM1 protein is associated with gastroesophageal adenocarcinoma (112) and elevated IFITM3 leads to unfavorable outcomes in acute myeloid leukemia (113). In addition, IFITM genes are highly expressed in human and murine colorectal tumors (114) with independent validation linking IFITM3 expression to colon carcinogenesis (115).

**Figure 2:**
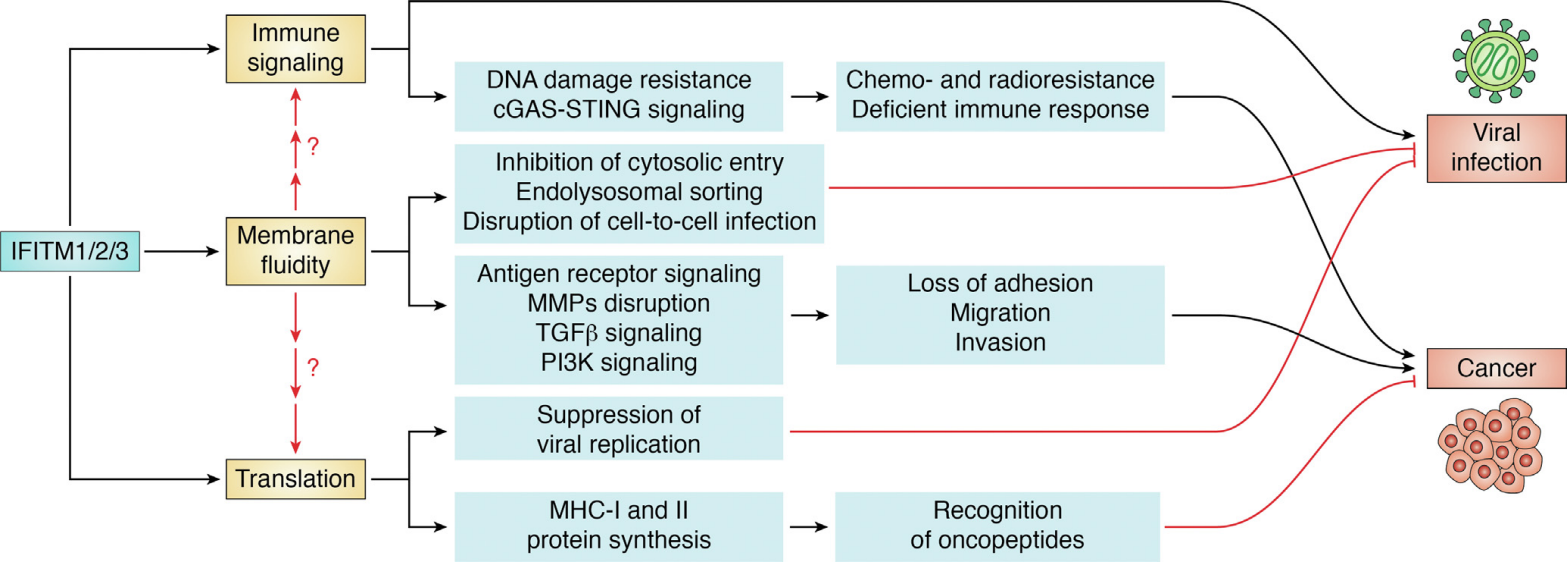
**An integrated model of IFITM function in viral infection, cancer, and immunity.** The three main mechanistic functions associated with IFITMs are shown, immune signaling, membrane fluidity, and translation. At present, the relationship between them is yet not well understood, though emerging membrane fluidity and curvature models in regards to viral infection could be central to all these phenomena. Such IFITM-mediated changes to membranes impair entry of viruses into the host cell. IFITMs also exhibit immunoregulatory effects, potentially including influence on cGAS-STING-IRF3 signaling upon infection and DNA damage in cancer. In addition, they may degrade extracellular matrix proteins and activate TGFβ and PI3K pathways, leading to metastasis of cancer cells, all of which may be conceivably influenced by IFITM-induced changes to membrane signaling platforms. Some studies have found a relationship between IFITMs and protein synthesis whereby they inhibit the production of viral proteins during infection, while in some cancers IFITMs increase the expression of MHC class I and MHC class II molecules, likely affecting the identification of oncopeptides.

Despite the frequently noted association of IFITMs with poor outcomes, the clinical relevance and molecular mechanisms of IFITM proteins in cancer generally remain poorly examined. Some studies have correlated the expression of IFITM3 in tumor tissue with cell growth, invasion, and metastasis (26, 109) and its loss with decrease of proliferation and promotion of cell arrest in G0/G1 phase (116). Moreover, one study reported that IFITM3 positively modulates activation of the TGFβ-MAPK-Smads pathway through an interaction with Smad4, thereby potentiating TGFβ-induced proliferation, cell migration, and invasion in bone metastasis (107). Interestingly, another study found that TGFβ stimulates the expression of IFITM3 in melanoma (117) and glioma cells (118). Wang *et al.* also observed that knockdown of IFITM3 repressed phosphorylation of the transcription factor STAT3 and reduced TGFβ-mediated invasion in a coculture model (Fig. 2).

Lee *et al.* have shown that B cells in leukemia and lymphoma also display elevated expression of IFITM3. These authors found that increased phosphorylation of IFITM3 Tyr20 in leukemic B cells anchors IFITM3 to the plasma membrane, and here IFITM3 acts to increase the activity of the oncogenic PI3K. Correspondingly, *Ifitm3*-deficient B cells have defects in B-cell receptor function and high affinity antibody formation. Moreover, reduced phosphatidylinositol(3,4,5)-trisphosphate (PIP3) levels following ablation of *Ifitm3* led to downregulation of over 60 lipid raft-associated cell surface receptors (including CD19) (75). It is possible this could also be in part due to the influence of the IFITM3 amphipathic helix on membrane fluidity (Fig. 2), although this possibility was not assessed. Notably, another study identified IFITM2 as a receptor for a secreted form of the chaperone BAG3, showing that IFITM2 mediated signaling through PI3K and p38 MAPK pathways (119) to promote tumorigenesis in a model of pancreatic ductal adenocarcinoma.

Other studies have proposed that IFITM1 negatively regulates the expression of Caveolin-1 (CAV1) and other proteins associated with cell migration and adhesion (106, 120). Silencing IFITM1 expression in glioma cells decreased proliferation and invasion processes by inducing cell cycle arrest in the G0-G1 phase. This also reduced the expression of the matrix metalloproteinase enzyme MMP9, which is associated with the initial stages of metastasis (121). Consistently, high expression of IFITM1 in head and neck squamous cell carcinoma correlates with upregulation of matrix metalloproteinases (122). Matrix metalloproteinases degrade extracellular matrix proteins, triggering cellular detachment from the local tumor microenvironment and ultimately allowing migration to distal sites (Fig. 2) (123).

The effects of IFITMs in cancer may appear to contrast with the proapoptotic, antiproliferative roles classically associated with IFN (124). However, the effects of IFN on cancer development are not uniform but rather dosage and tissue context dependent. For example, Benci *et al.* (125) found, in contrast to what is seen with whole body IFNAR KO models, transplanted IFNAR KO tumors grew less aggressively than WT *in vivo*. Thus, in this context, the overall effect of tumor intrinsic IFN signaling was to favor tumor development. In other contexts, the cytotoxic anticancer effects of IFN are demonstrable in cancer cells (43, 44). Type I IFNs have been shown to induce DNA repair activities in multiple systems—indeed, multiple ISGs directly promote DNA repair including SAMHD1, ISG15, and PARP9 (126, 127, 128)—and at low doses these activities dominate the cytotoxic activities (129, 130). Interestingly, a recent report found that siRNA knockdown of IFITM1 sensitized oral squamous cell carcinoma cells to ionizing radiation (131). It could be that IFITM1 is directly involved in DNA repair, although it is not clear by what mechanism this could occur.

**Figure 3:**
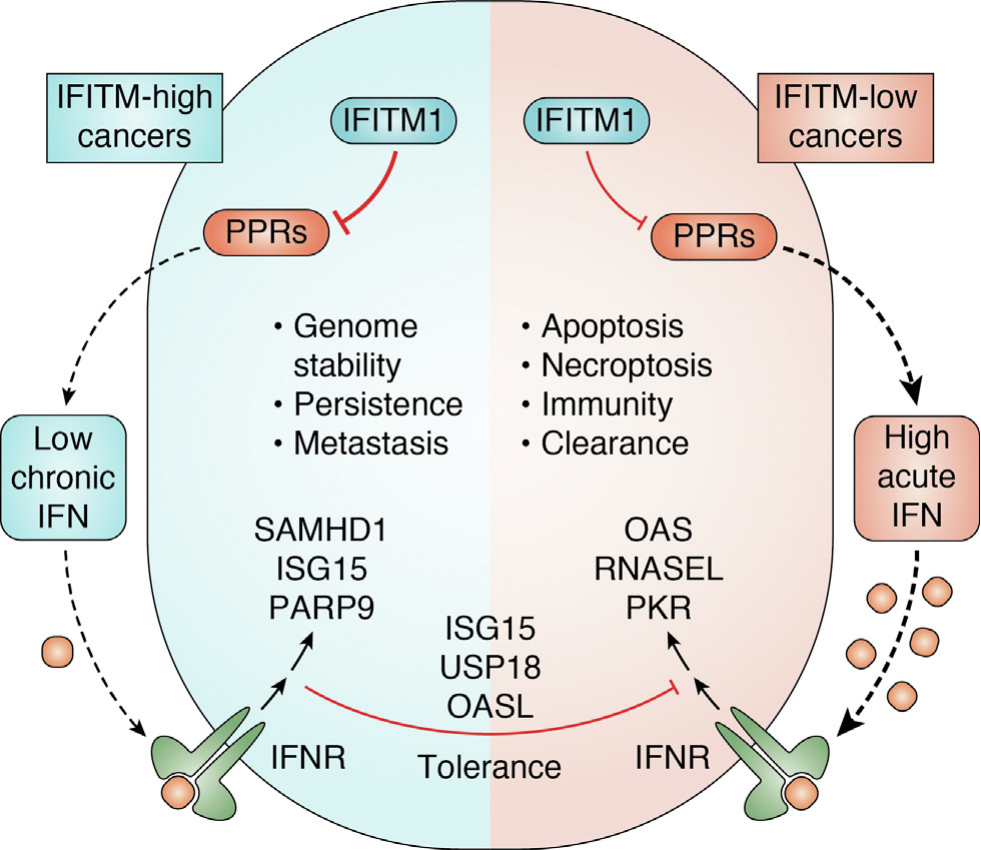
**The effects of IFITM proteins on type I IFN signaling in cancer.** In cancer, radiation and chemotherapy along with other sources of DNA damage are ultimately sensed by pattern recognition receptors (PRRs) that activate the type I IFN response. A chronic low level of type I IFN (*blue box*) promotes protumor effects, whereas acute high levels of type I IFN (*red box*) promote a protective immune response toward cancer. We propose a speculative model whereby IFITMs create a negative feedback loop to repress the effects of ongoing PPR stimulation and create a state of type I IFN resistance in cancer.

Several ISGs have been implicated in cancer progression by mechanisms that do not relate directly to DNA repair. The RNA deaminase ADAR1 is an ISG, which mediates resistance to immune checkpoint blockade in cancer (46). It does this by preventing the activation of RIG-I like receptors by self RNA, thereby limiting the induction of mitochondrial antiviral-signaling protein-dependent cytokine responses (132). The nuclease TREX1 is another ISG, which limits tumor immune activation (48). This occurs because of its ability to degrade cytosolic DNA, which appears in cancer cells with high levels of DNA damage, thereby preventing activation of the cGAS-STING signaling pathway (133). As discussed in the preceding section, multiple nascent strands of evidence support that IFITMs may negatively regulate activation of innate immune pathways, although to date most work has focused on IFITM3. It is interesting to consider whether “anti-inflammatory” mechanisms like those exhibited by ADAR1 and TREX1 could explain some of the observations in cancer patients with elevated expression of IFITMs. This would have the effect of reducing activation of peripheral immunity and shifting the IFN milieu toward a prorepair, noncytotoxic equilibrium (Fig. 3).

## Overlaps between the role of IFITM proteins in viral infection and in cancer

A growing body of evidence has defined IFITMs both as restriction factors and protumor factors. The association perhaps is not surprising given the many molecular and immunological commonalities between virus-infected and malignant cells. Viruses and malignant cells trigger the activation of the immune system and under some circumstances, take advantage of depressed host immunity (134, 135). As has been mentioned already, IFITMs are not the only proteins exhibiting this dual status; the ubiquitin-like protein ISG15 is another example. ISG15 is rapidly induced by IFN and capable of modifying a substantial number of proteins by an ubiquitin-like protein modification termed ISGylation. USP18, UBE1L, and p53 are examples of ISG15-conjugated proteins that are linked to cancer. However, the literature is mixed as to whether ISG15 has protective or adverse effects during cancer, likely because ISG15 is able to modify a vast number of proteins, resulting in opposing effects depending on context. ISG15 is additionally implicated in innate immune antiviral responses and can inhibit viral replication (136).

Both the virus infected and the cancerous cellular states compromise cellular viability by manipulating the cell cycle and hijacking the translation and proteostasis systems of the cell for their own convenience (137, 138, 139). Recent work suggests that IFITMs have a regulatory role in mRNA translation. IFITMs suppress viral replication by selectively excluding HIV-1 mRNA transcripts from polysomes (24). Consequently, the synthesis of viral proteins is decreased, resulting in low virion production. Translational inhibition could be reversed by codon optimization of HIV-1 *gag*, suggesting that recognition of viral RNA was linked to the phenotype. Similarly, another study showed that IFITM-mediated inhibition of HIV-1 production was sensitive to viral dsRNA structural segments (23).

In the context of cancer, KO of IFITM1/3 impairs the protein synthesis of a group of molecules induced by IFNγ including HLA-B and ISG15. HLA-B belongs to the MHC class I complex and it is a key component of the antigen-presentation pathway (Figs. 1 and 2) (140). Reducing MHC class I expression disrupts the production, processing, and presentation on the cell surface of novel epitopes by the antigen-presentation machinery, impairing CD8+ T-cell recognition (141, 142, 143) (Fig. 1). In addition, downregulation of IFITM3 also decreases transcription of MHC class II and components of the complement cascade (144). This trait confers a positive advantage by reducing tumor cell recognition over other tumor variants. To counteract this effect, cancers and viruses suppress MHC signaling (145, 146).

Further work on the impact of IFITM proteins in the context of cancer revealed that IFITM proteins may even *directly* influence translation. IFITM1 was found to interact with the cytoplasmic splicing factor protein SRSF1 in a cervical carcinoma cell line and was found to directly interact with HLA-B mRNA (147). Furthermore, IFITM1/IFITM3 double KO cells had a reduced 80S ribosomal fraction, while IFITM proteins could be detected in polysomes. Notably, genetic KO of IFITM1 and IFITM3 inhibited the synthesis of a subset of IFN-responsive proteins, which is consistent with the notion of an immunoregulatory role for IFITMs in cancer (Fig. 3).

Taking these studies together, it seems clear that IFITM proteins regulate translation in some manner, both promoting and repressing protein synthesis, depending upon context. It is notable that these unrelated studies using different cell models converged on the same novel mode of action for IFITMs. IFITM proteins had not previously been implicated in a translational role. In addition to their well-accepted localization on the cell surface or in endosomal compartments, IFITM3 mutants lacking cysteine residues that are sites of palmitoyl modifications were found in the endoplasmic reticulum (148, 149). Better definition of this perinuclear localization and its relationship to the rough endoplasmic reticulum might be of value in understanding the role IFITM3 plays in regulation of translation. Notably, IFITM3-STING interactions seemed to occur here also (86). Ribosomes can exchange readily between the endoplasmic reticulum and the cytosol, with endoplasmic reticulum docked ribosomes able to access the full pool of cytosolic mRNAs following at most a few rounds of translation at the endoplasmic reticulum (150, 151), which could potentially explain how membrane proteins affect the translation of nonmembrane proteins such as HIV-1 Gag or ISG15.

Previous publications have pointed out that posttranslational modifications of IFITMs regulate their subcellular location and as a result profoundly influence their function (149, 152, 153). For instance, phosphorylation of Tyr20 residue forces IFITM3 to reside at the cell membrane, switching from antiviral properties in the endosomes to function as an oncogenic receptor (75). Although upregulation of IFITM3 on the cell surface reduces protection against IAV (21), it enhances anti-HIV activity by more potently restricting viral entry (154). IFITM3 is present in multiple organelles, and internalization into late endosomes, multivesicular bodies, and lysosomes could potentially affect its function. Considering IFITMs as antiviral proteins, the tendency is to think of them simply as membrane proteins waiting at sites of viral entry. Whether ‘mislocalized’ IFITM not present at these sites is alternatively functional is not yet clear, but given the range of described alternative functions, this remains a possibility that should be investigated. This combined with the potential for a degree of redundancy between the proteins may complicate their future study.

## Concluding remarks

IFITM proteins are well-defined antiviral effectors that restrict viral entry through disruption of membrane properties. Studies indicating how this proceeds mechanistically have recently emerged. However, there are many reports of functions of IFITMs that seem to fall outwith this particular non-antiviral modality. For example, IFITM proteins have been variously implicated in viral translation, vesicle trafficking, nuclear translocation, and proteolytic processing. Beyond this, there are suggested nonantiviral functions involving lipid raft composition, receptor signaling, vesicle trafficking, and immune regulation. Collectively, the multiple cellular roles of IFITMs uncovered by virologists, cancer biologists, and immunologists parallel the diversity of physiological and disease contexts in which IFITMs are implicated (Fig. 1). The clear importance of IFITM proteins both in *in vivo* models and in a range of clinical contexts creates an imperative for resolving these varied observations.

### What questions do we need to ask next?

Many cellular processes are impacted by lipid membrane dynamics, and the lead mechanistic model for the antiviral activity of IFITMs concerns their regulation of membrane dynamics. Thus, it is reasonable to ask whether this may influence observed phenotypes beyond viral entry restriction. An important study in this regard is that of Lee (75), which showed that IFITM3 expression disrupted membrane lipid rafts, leading to reductions in expression of multiple cell surface receptors and the loss of PI3K signaling and B-cell receptor signaling. It is possible that IFITM-mediated disruption of cell surface receptors and lipid rafts could also underpin some of the IFITM-dependent behaviors observed in other studies. For example, IFITM3 is proposed to increase TLR2 surface levels through regulation Nogo-B, but it is interesting to consider how this relationship could be impacted by IFITM-mediated membrane disruption (85). Unpicking the role of IFITM proteins may be challenging if there is systemic disruption of cell surface receptor signaling, but there is value in considering prior conclusions with this paradigm in mind (Fig. 3).

Membranes are more than just signaling platforms; they have key roles in internal and external vesicle trafficking, as well as maintaining cellular integrity. It has been shown that IFITM3 affects endocytic vesicle maturation (16), and it is suggested that under some circumstances, IFITM3 expression influences Golgi trafficking of glycoproteins (155). Could these influences on trafficking also be driven by localized disruption of membrane curvature or fluidity? Much would depend on IFITM localization. IFITM proteins can be frequently seen in locales beyond the cell surface and endosomes and a fuller context-dependent account is needed. Given that IFITMs have been shown to affect a range of membrane-dependent processes, including fusion, signaling, and vesicle trafficking, it is conceivable that other aspects of membrane biology could be similarly affected. This will again depend on detailed accounts of localization, which are often constrained due to the use of IFITM overexpression or through antibody crossreactivity between IFITMs.

More recently, IFITM3 has been described to bind ɣ-secretases and modify their function, leading to increased amyloid-β production (156). In mice, IFITM3 KO reduced amyloid plaque formation, while enhanced association between IFITM3 and ɣ-secretase complexes were noted in patients with late onset Alzheimer's disease. Secretases are lipid raft proteins and their function has been described to be cholesterol dependent in some instances (157); it may then be possible that IFITM3 driven changes to lipid raft fluidity or membrane curvature could influence ɣ-secretase activity. Though it may also be that systemic disruption of lipid raft proteins and function (75) or modulation of protein function by direct protein–protein interactions, drive the phenotype.

We have also described a role for IFITMs in regulating mRNA translation in both viral infections and in cancer cells. Again, how much of this could be ascribed to a model of disrupted membrane function? It is conceivable, for example, that IFITMs could act at the endoplasmic reticulum membrane to influence the translation of transmembrane proteins such as HLA-B (or likewise, secretory proteins) by indirectly perturbing the Sec61–ribosome complex. However, surprisingly, translation of nonmembrane proteins such as HIV-1 Gag and ISG15 was also found to be influenced by IFITM expression. It could be that the initial influence of IFITMs on translation at the endoplasmic reticulum has wider consequences when ribosomes exchange back to the cytosol, as has previously been observed in other contexts (150, 151). Further studies are needed to determine the mechanism by which IFITMs regulate protein synthesis; in particular, whether IFITMs form part of the translation machinery in ribosomes (cytosolic or Sec61 associated) or if they regulate mRNA transport to ribosomes.

Despite the appeal of a single unifying mechanism that ties together many of the observations in this review (Fig. 2), this need not be the case. For example, in instances where IFITM proteins are found to have direct protein–protein interactions, membrane disruption hypotheses may be harder to invoke or at least not provide a full account of IFITM function, such as with SARS-CoV-2 Spike, HIV-1 Env, IAV HA, ɣ-secretases, BAG3, or Nogo-B (62, 64, 85). However, given that a consensus regarding IFITM antiviral function is emerging regarding membrane fluidity or curvature, it will be important to address this mechanism, even when direct protein–protein interactions are noted. In the case of both receptor signaling and translation, it may be possible to use new mechanistic insights on the function of the IFITM amphipathic helix to create separation-of-function mutants that will allow the discrimination of membrane fluidity effects from more direct protein–protein interaction effects such as these.

### Unifying understanding of IFITMs and IFITM research

IFITM research to date has been somewhat segregated. On the whole, viral IFITM research has pursued virus entry as a paradigm. This has been a high value endeavor that has brought forward a degree of mechanistic and clinical understanding. However, there is a need to move beyond this exclusive understanding because IFITMs have pleiotropic effects that extend to cellular function and immune regulation. In particular, *in vivo* experiments need to be planned and interpreted in the context of wider disruption on cells and immunity. There may be a need to revisit our interpretations of some such studies in the light of these noncanonical influences of IFITM proteins (Fig. 2).

In the area of cancer research (Fig. 3), there might be value in engagement with the mechanistic models that have emerged through viral research, and both cancer and virology fields need to consider the emerging role of IFITMs in immune regulation. A priority will be to uncover why some immune regulatory phenotypes are opposing. It is also becoming apparent that IFITMs may have a more general role in influencing membrane vesicles and protein trafficking in the cell. Continuing to address these phenomena and other membrane processes may help to reveal a more fundamental cellular role for IFITM proteins; it is likely that not all IFITM phenotypes described will fall within membrane shaping and fluidity models. Some of these may be accounted for due to the wide-ranging influence of disrupting innate immune signaling or lipid raft signaling platforms but being open to the possibility of further IFITM functions is clearly warranted.

A way forward may be through increased use of unbiased methodologies in IFITM research. Beyond their description as antiviral proteins (18, 158, 159), there have been relatively few such studies. Three different affinity proteomic studies found different sets of IFITM interacting proteins with minimal overlap (85, 87, 147). Whether this is due to technical artefact or context specific IFITM function is not yet known. Thus, a more systematic and context-dependent account of IFITM proteins will be of value.

### Perspectives

IFITMs have been intensively studied in recent years. This has not been undue; their biological and clinical importance is underscored by their emergence as a marker of therapeutically resistant and aggressive cancers, with *in vivo* and human genetics studies linking them to viral control and inflammatory diseases. This diversity of study should be considered a strength, as there is significant potential for a range of diverse fields to benefit from each other’s understanding of IFITMs. Given the clear importance of IFITMs *in vivo* and that they appear to be druggable in principle (58, 160, 161, 162, 163), developing a more integrated understanding of IFITMs will be a high yield endeavor.

## Conflict of interest

The authors declare that they have no conflicts of interest with the contents of this article.
